# Evolution and maintenance of a large multidrug-resistant plasmid in a *Salmonella enterica* Typhimurium host under differing antibiotic selection pressures

**DOI:** 10.1128/msystems.01197-24

**Published:** 2024-10-22

**Authors:** Ming Cheng, Jing-Jing Dai, Jin-Fei Zhang, Yu-Ting Su, Si-Qi Guo, Ruan-Yang Sun, Dong Wang, Jian Sun, Xiao-Ping Liao, Sheng Chen, Liang-Xing Fang

**Affiliations:** 1 Guangdong Laboratory for Lingnan Modern Agriculture, National Risk Assessment Laboratory for Antimicrobial Resistance of Animal Original Bacteria, College of Veterinary Medicine, South China Agricultural University, Guangzhou, Guangdong, China; 2 Guangdong Provincial Key Laboratory of Veterinary Pharmaceutics Development and Safety Evaluation, South China Agricultural University, Guangzhou, Guangdong, China; 3 Department of Food Science and Nutrition, Faculty of Science, The Hong Kong Polytechnic University, Kowloon, Hong Kong, China; Marquette University, Milwaukee, Wisconsin, USA

**Keywords:** plasmid, fitness cost, antibiotic resistance, experimental evolution, positive selection, loss of plasmid-encoded ARGs

## Abstract

The dissemination of antibiotic resistance genes (ARGs) through plasmids is a major mechanism for the development of bacterial antimicrobial resistance. The adaptation and evolution mechanisms of multidrug-resistant (MDR) plasmids with their hosts are not fully understood. Herein, we conducted experimental evolution of a 244 kb MDR plasmid (pJXP9) under various conditions including no antibiotics and mono- or combinational drug treatments of colistin (CS), cefotaxime (CTX), and ciprofloxacin (CIP). Our results showed that long-term with or without positive selections for pJXP9, spanning approximately 600 generations, led to modifications of the plasmid-encoded MDR and conjugative transfer regions. These modifications could mitigate the fitness cost of plasmid carriage and enhance plasmid maintenance. The extent of plasmid modifications and the evolution of plasmid-encoded antibiotic resistance depended on treatment type, particularly the drug class and duration of exposure. Interestingly, prolonged exposure to mono- and combinational drugs of CS and CIP resulted in a substantial loss of the plasmid-encoded MDR region and antibiotic resistance, comparable to the selection condition without antibiotic. By contrast, combinational treatment with CTX contributed to the maintenance of the MDR region over a long period of time. Furthermore, drug selection was able to maintain and even amplify the corresponding plasmid-encoded ARGs, with co-selection of ARGs in the adjacent regions. In addition, parallel mutations in chromosomal *arcA* were also found to be associated with pJXP9 plasmid carriage among endpoint-evolved clones from diverse treatments. Meanwhile, *arcA* deletion improved the persistence of pJXP9 plasmid without drugs. Overall, our findings indicated that plasmid-borne MDR region deletion and chromosomal *arcA* inactivation mutation jointly contributed to co-adaptation and co-evolution between MDR IncHI2 plasmid and *Salmonella* Typhimurium under different drug selection pressure.

**IMPORTANCE:**

The plasmid-mediated dissemination of antibiotic resistance genes has become a significant concern for human health, even though the carriage of multidrug-resistant (MDR) plasmids is frequently associated with fitness costs for the bacterial host. However, the mechanisms by which MDR plasmids and bacterial pairs evolve plasmid-mediated antibiotic resistance in the presence of antibiotic selections are not fully understood. Herein, we conducted an experimental evolution of a large multidrug-resistant plasmid in a *Salmonella enterica* Typhimurium host under single and combinatorial drug selection pressures. Our results show the adaptive evolution of plasmid-encoded antibiotic resistance through alterations of the MDR region in the plasmid, in particular substantial loss of the MDR region, in response to different positive selections, especially mono- and combinational drugs of colistin and ciprofloxacin. In addition, strong parallel mutations in chromosomal *arcA* were associated with pJXP9 carriage in *Salmonella* Typhimurium from diverse treatments. Our results thus highlight promoting the loss of the plasmid’s MDR region could offer an alternative approach for combating plasmid-encoded antibiotic resistance.

## INTRODUCTION

Plasmid-mediated horizontal gene transfer plays an essential role in bacterial genome evolution, especially in terms of the global spread of antibiotic resistance, which constitutes a major threat to public health. In particular, conjugative multidrug-resistance (MDR) plasmids possessing antibiotic resistance genes (ARGs) against multiple antibiotic classes are of particular concern as they allow the instantaneous acquisition of MDR phenotypes and thus potentiate the rapid emergence of MDR bacteria. Therefore, understanding the mechanisms that underlie the coevolution and coadaptation of an MDR plasmid and the bacterial host is crucial to predict the prevalence of host-plasmid associations. This is particularly important for epidemic high-risk clones and the evolution of plasmid-mediated antibiotic resistance mechanisms.

Plasmids provide bacteria with adaptive survival tools even though their presence frequently incurs fitness cost on the bacterial host in the absence of selection for plasmid-encoded traits (1). Importantly, these costs can act as a major barrier for plasmid maintenance, although compensatory mutations in either host or plasmid genes may occur that facilitate plasmid carriage. This has been demonstrated during evolution experiments using co-culture of plasmid and bacteria in three cases. First, it relates to the case of small, multicopy, and non-conjugative plasmids in the absence of antibiotic exposure, plasmid-host co-adaptations mostly occurred via mutations in the plasmid replication machinery (2, 3), in plasmid-encoded genes that directly altered bacterial growth (2) and in chromosomal genes interacting with replication proteins (4). Second, it relates to the case of large, low-copy-number plasmids in the absence of antibiotic exposure, and deletions of plasmid MDR and conjugation transfer regions are frequently encountered for plasmid-host co-adaptations (5, 6). Besides plasmid evolution, chromosomal compensatory mutations have been found in global regulatory systems such as GacA/GacS (secondary metabolism), carbon catabolite repression, ArcAB (aerobic respiration control), and PFLU4242 (unknown function, possibly related to partitioning systems) (7, 8). Third, it relates to the case of resistance or MDR plasmids in the presence of antibiotic exposure, and compensatory mutations were also involved in plasmid-host co-adaptations (9–13). For example, *Escherichia coli* possessing a tetracycline (TET) resistance plasmid culture in the presence of TET-generated adaptive mutations in host and plasmid genomes in an ordered and highly repeatable manner across independently evolving populations (12). Furthermore, the use of an MDR plasmid in *E. coli* under both TET and TET/ampicillin combinations resulted in elevated TET resistance albeit at reduced fitness costs that had occurred via intragenomic coevolution (13). However, the evolution of large MDR plasmids and plasmid-mediated antibiotic resistance in bacterial hosts has been less studied under diverse antibiotic selection pressures.

IncHI2 plasmids are widespread among Enterobacteriaceae and are commonly large (>200 kb) and share conserved backbone structures but have distinctively variable regions containing multiple ARGs. In particular, IncHI2 plasmids are particularly prevalent in the zoonotic pathogen *Salmonella* spp. Ours and other previous epidemiological studies have demonstrated that IncHI2 plasmids frequently co-spread multiple and clinically important ARGs including plasmid-mediated colistin (CS)-resistance gene *mcr-1*, extended spectrum beta-lactamases gene *bla*_CTX-M_, and plasmid-mediated quinolone resistance genes *oqxAB* among *Salmonella enterica* especially Typhimurium and its variant 1,4,[5],12:i:- from both food animals and humans in China (14–17) and other countries (18). The *bla*_CTX-M-14_ gene confers resistance to third-generation cephalosporins, and the *oqxAB* genes reduce resistance to quinolones in Enterobacteriaceae. It is notable that both fluoroquinolones and extended-spectrum cephalosporins are used as front-line antibiotics for treating gram-negative bacterium bacterial infections, including *Salmonella*, in humans. Furthermore, the *mcr-1* gene confers resistance to colistin, which is used as a last-resort antibiotic for the treatment of MDR gram-negative bacterial infections. Thus, understanding the mechanism underlying the co-adaptation and co-evolution between MDR IncHI2 plasmid and its bacterial host might explain the widespread distribution and stable maintenance of IncHI2 plasmid among *Salmonella spp*. and help to impede the emergence and spread of successful bacteria-plasmid associations, as well as prevent salmonellosis in humans.

In the current study, we focused on the IncHI2 plasmid pJXP9 (244 kb), which has a typical IncHI2 plasmid backbone structure and carried 15 ARGs including *mcr-1*, *bla*_CTX-M-14_, and *oqxAB*. We experimentally evolved *S*. Typhimurium ATCC 14028 carrying the MDR plasmid pJXP9 under a range of antibiotic treatment regimens including no antibiotic, mono-, and combination-treatments of CS, cefotaxime (CTX), and ciprofloxacin (CIP). Following ~600 generations of selection, we quantified evolved changes in plasmid-mediated antibiotic resistance and fitness and used genome sequencing to determine the dynamic and evolution of plasmid pJXP9.

## MATERIALS AND METHODS

### Strains and experimental evolution

The *S*. Typhimurium strain 14028-pJXP9 is a transconjugant obtained by transferring plasmid pJXP9 into 14028 via conjugation as previously described (19). Plasmid pJXP9 is derived from *S*. 1,4,[5],12:i:- JXP9 strain and carried 15 ARGs including *mcr-1*, *bla*_CTX-M-14_, *oqxAB*, *fosA3*, and *floR* (14). Strain 14028 and 14028-pJXP9 were used as the initial ancestral strains for *in vitro* laboratory evolution experiments. Briefly, cultures were grown in 5 mL Luria Bertani (LB) broth in 50 mL tubes at 37°C and shaken at 180 rpm. Independent selection lines of strain 14028-pJXP9 were found using 42 independent single colonies of 14028-pJXP9 taken from plate cultures and split into 14 exposure groups with biological triplicates as follows: mono drug exposures (i) CS-low/middle/high groups: 0.125 (1/128 minimum inhibitory concentration [MIC]), 1 (1/16 MIC), and 8 (1/2 MIC) μg/mL; (ii) CTX-low/middle/high groups: 1 (1/128 MIC), 8 (1/16 MIC), and 64 (1/2 MIC) μg/mL; (iii) CIP-low/middle/high groups: 0.001 (1/128 MIC), 0.008 (1/16 MIC), and 0.06 (1/2 MIC) μg/mL; combinational drug exposures (i) CTX-CIP: 1 µg/mL CTX (1/128 MIC) and 0.015 µg/mL CIP (1/8 MIC); (ii) CTX-CS: 1 µg/mL CTX and 2 µg/mL CS (1/8 MIC); (iii) CIP-CS: 0.015 µg/mL CIP and 2 µg/mL CS; (iv) CTX-CIP-CS: 1 µg/mL CTX, 0.015 µg/mL CIP, and 2 µg/mL CS; no-drug exposure. In parallel, three independent 14028 colonies without drug pressure were used as the control group. Cultures were serially diluted 1/1,000 every consecutive day (24 h) for 63 days to achieve ~600 generations calculated as previously described (19). The final tally for these experiments was 45 endpoint-evolved populations (14 exposure scenarios, 1 control group, and biological triplicates for each group), which were stored in 30% glycerol at −80°C.

### Antibiotic susceptibility testing, ARG detection, and plasmid stability

Plasmid-mediated antimicrobial susceptibilities were determined among the selected endpoint-evolved clones by agar dilution (including CTX, CIP, florfenicol [FFC], fosfomycin [FOS], and nalidixic acid [NAL]) or broth microdilution methods (CS), and the results were interpreted according to the Clinical and Laboratory Standards Institute (CLSI) (20) and veterinary CLSI (21). In addition, the MIC for FOS was determined using Mueller Hinton agar supplemented with 25 µg/mL glucose-6-phosphate. *E. coli* ATCC 25922 was used as the quality control strain. The presence of *mcr-1*, *bla*_CTX-M-14_, *fosA3*, *oqxAB*, and *floR* carried by pJXP9 among the selected endpoint-evolved clones was determined using PCR as previously (19). To determine the maintenance of plasmid pJXP9 among endpoint-evolved populations, the presence of the replication initiation protein gene *repHI2* was determined using PCR as previously described (22).

### Genome sequencing analysis

Whole-genome sequencing (WGS) was performed among the endpoint-evolved 14028-pJXP9 clones and the evolved populations. Genomic DNA was extracted using Gentra Puregene bacterial DNA purification kit (Qiagen, Hilden, Germany) according to the manufacturer’s instructions. The genomic DNA was sequenced using an Illumina HiSeq platform (Novogene, China), and sufficient sequencing depth (>100) was obtained for further analysis (1 Gb per clone and 6 Gb per population). The raw data of genomes were filtered by Trimmomatic v0.32 with -phred 33. For clones, the filtered reads were assembled using SPAdes v3.6.2 (-t 30 k 21,33,55,77,99,127 --careful --phred-offset 33) (23). Sequencing quality and statistics per isolates were checked using the Quast v5.2.0 (Table S1) (24).

Sequencing reads of endpoint-evolved clones were mapped to the ATCC 14028 reference genome (CP001363), and basic variants were called using the CLC Genomics Workbench 10.0 (Qiagen, Hilden, Germany) with default parameters (basic variant detection, similarity fraction of 0.9, minimum coverage of 100, minimum count of 10, and minimum frequency of 10.0%) as previously described (19). All chromosomal mutations occurring in 10% of the reads and in at least 10 unique reads were included in the analysis, while those occurring in noncoding regions were excluded. Then, the mutations that were present in corresponding endpoint-evolved 14028 clones were filtered out from the above-described mutation results.

Alignments between evolved pJXP9 plasmids from clones and the ancestral pJXP9 plasmid were conducted, and the generation of circular maps was constructed using BRIG v0.95 (25). For populations, sequencing reads were mapped to the plasmid pJXP9 sequence (Acc. No. MK673549) using SOAP aligner/SOAP2 (v2.20 with options: -M 4 [find best hits], -l 30 [seed length], -r 1 [random assignment of multiple hits], -v 5 [maximum number of mismatches], and -o PE_output [output file for alignment results]). Highly mapped reads were subsequently filtered using a 30 bp length and 95% identity cutoff, and gene-length normalized base counts were calculated using the soap.coverage script (https://github.com/gigascience/bgi-soap2/tree/master/tools/soap.coverage/2.7.7) (26). The gene relative abundance of pJXP9 (all genes in pJXP9) from endpoint and mid-point evolved populations was normalized using RPKM (reads per kilobase per million reads) with pJXP9 from ancestral 14028-pJXP9 populations as the reference as previously described (19, 27), and generation of synteny plots was constructed using Prism. To confirm the sizes of pJXP9 in endpoint-evolved 14028-pJXP9 clones, S1-PFGE and Southern blotting were carried out as previously described (19).

### Growth curves and competition experiment *in vitro*

The growth and competition assays were conducted as previously described (19). Briefly, growth curves were measured using triplicate overnight culture that was diluted to the optical density at 600 nm (OD_600_) = 0.1 in phosphate-buffered saline (PBS), and 200 µL dilutions were diluted into 20 mL LB broth in conical flask (100 mL) at 37°C with 180 rpm shaking. Then, 200 µL bacterial culture was transferred into a 96-well flat-bottom plate every hour during the culture process, and blank LB broth was used as a negative control. Growth curves were determined by measuring OD_600_ using a multimode reader plater Ensight (PerkinElmer, USA). The aliquots were collected from 0 to 12 h at every hour. The area under the growth curve (AUC) was determined by using the Growthcurver package in R. The maximum growth Rate (estimating the intrinsic population growth rate and defining as the linear slope of the tangent line passing around the time of maximum growth) and the Lag Time (defining as the integrated time lost during the adaptation to new conditions compared with an immediate response) were determined by using OriginLab 2021 (28, 29).

The relative fitness (RF) of endpoint-evolved 14028-pJXP9 clones vs ancestral 14028-pJXP9 was determined by using *in vitro* competition assays. In brief, growth competition was initiated using strain cultured overnight in LB medium at 37°C, then diluted to OD_600_ = 0.1, mixed in 5 mL LB broth at a ratio of 1:1, and incubated at 37°C. Cultures were transferred into 5 mL fresh LB broth every 24 h for 3 days. The cultures at 0, 24, 48, and 72 h were individually diluted to obtain separated colonies using LB agar containing cefotaxime/colistin/florfenicol or not. Considering it could not distinguish some endpoint-evolved 14028-pJXP9 clones and ancestral 14028-pJXP9 by plating the diluents on LB agar containing antibiotic. In those cases, we used 14028 as the competitor against these endpoint-evolved 14028-pJXP9 clones and ancestral 14028-pJXP9, respectively (30). The relative fitness was calculated as follows: RF = (log_10_ S1dt − log_10_ S1d0)/(log_10_ S2dt − log_10_ S2d0), where S1 and S2 represent colony-forming units of two tested clones (*t* = time in days), respectively. RF > 1 indicated a selective advantage over the control strain, whereas RF < 1 represented a fitness cost.

### Conjugation experiments *in vitro*

Conjugation experiments were performed by solid mating using endpoint-evolved 14028-pJXP9 clones as donors and the 14028-rif (ancestral *S*. Typhimurium ATCC14028 acquired rifampicin [RIF] resistance) as recipients as previously described (30). Transconjugants carrying evolved pJXP9 were selected on LB agar plates supplemented with RIF (50 µg/mL) and CTX (4 µg/mL) or CS (2 µg/mL). Transfer of antibiotic resistance phenotype and genotypes was determined using antimicrobial susceptibility testing and PCR, respectively, as described above. 14028-rif-pJXP9 transconjugateswere also submitted for WGS as described. Growth curves and competitive fitness assays were performed among 14028-rif-evolved pJXP9 transconjugants compared to 14028-rif-pJXP9 as described above.

### Function verification

The selected target gene *arcA* was deleted in ancestral 14028 and 14028-pJXP9 by using the two-plasmid system pCasKP-pSGKp and pCasPA-pSGKp. The designed 20-nt base-pairing region (N20) of small guide RNA (sgRNA) for deleting targeted gene and primers for detecting fragment deletion are listed in Table S2 and based on CRISPR/Cas9-mediated genome editing method (31). Growth curves and competitive fitness assays were performed to compare the ancestral strains 14028∆*arcA* and 14028∆*arcA*-pJXP9 with 14028 and 14028-pJXP9, respectively, as described above. The stability of pJXP9 in 14028 and 14028∆*arcA* was investigated *in vitro* according to a previously described protocol with minor modification (32). Briefly, 14028∆*arcA*-pJXP9 and 14028-pJXP9 were propagated by serial transfer for 63 days in triplicate and then each culture broth was serially diluted in 0.9% saline and plated onto LB agar lacking antibiotics. Approximately 50 colonies were randomly chosen from each plate to confirm the presence of the *repHI2* replicon type by PCR assay as previously (22).

### Statistical analyses

Statistical analyses were performed using SPSS Statistics 27 (IBM, Armonk, New York, USA) and Prism 8.0 (GraphPad, San Diego, CA, USA). Spearman correlation coefficient was analyzed by an online platform (https://www.bioinformatics.com.cn).

## RESULTS

### Plasmid-encoded MDR regions and ARGs are largely altered in endpoint-evolved populations under different positive selections

**Fig 1:**
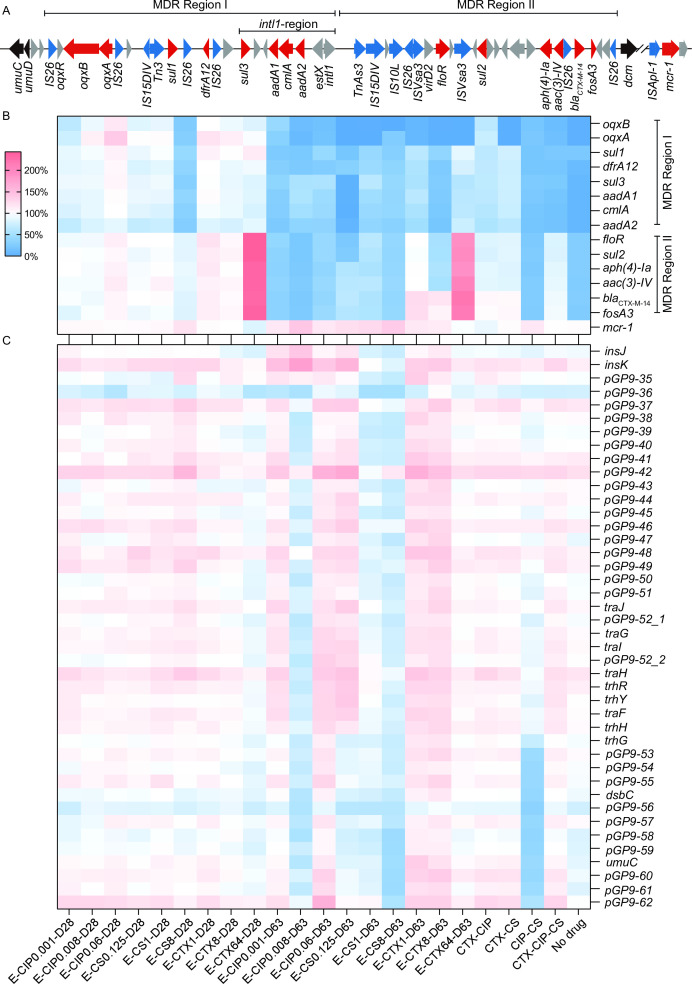
Characteristics of the gene relative abundance of ARGs carried by pJXP9. (**A**) The genetic contexts of ARGs carried by pJXP9. (**B**)The gene relative abundance of ARGs and (**C**) conjugative transfer region I carried by pJXP9 from the mid-point (transfer day 28) and endpoint (transfer day 63) evolved populations.

Initially, we examined the evolutionary dynamics of plasmid pJXP9 and plasmid-mediated antibiotic resistance in *Salmonella*. The bacteria were cultivated under mono-drug treatments for CS, CTX, and CIP at three different antibiotic concentrations. To analyze the changes, we compared the gene relative abundance in pJXP9 from nine mid-point populations (at transfer 28) and nine endpoint populations (at transfer 63) to the ancestral populations using WGS analysis. The results revealed that, overall, the gene relative abundance in pJXP9 from the mid-point-evolved populations did not significantly change for all the single antibiotic treatments, except for the CTX-high and CS-high groups. In the CTX-high group, the gene relative abundance sharply increased to 200%–310% for the MDR region II (D2, approximately 25 kb), which contained *bla*_CTX-M-14_, *fosA3*, and *floR* between *intl1* and *dcm*. On the other hand, in the CS-high group, the gene relative abundance sharply decreased to 30%–40% for most genes in the MDR region I (D1, approximately 26 kb), which contained *oqxAB* between *pGP9-62* and *pGP9-69* (Fig. 1A and B; Fig. S1; Data S1).

The populations that evolved at the endpoint displayed different patterns under single antibiotic treatments. Exposure to CIP resulted in decreases of 10%–60% in gene relative abundance in the MDR region between *pGP9-62* and *dcm* (~51 kb), which included D2 and D1. Additionally, gene abundance slightly decreased and was retained at levels of 60%–80% for the conjugative transfer region I (C1, ~40 kb) from the CIP-middle group. In contrast, there were slight increases in gene abundance for almost all pJXP9-encoded genes, except for the MDR region and C1, which were most evident in the CIP-high group (Fig. 1; Fig. S1; Data S2).

Exposure to CS resulted in a sharp decrease in ARG levels (to 0%–65%) across the MDR region. Particularly, *oqxAB* levels were near the detection limit. Similarly, the CS-low group displayed gene levels near the detection limit for the 10.069 kb fragment containing *intl1-estX-aadA2-cmlA-aadA1-sul3* (*intl1*-region) located on MDR region I. Furthermore, in the CS-high group, gene relative abundance slightly increased in the regions between *dcm* and *insAB*, which included *mcr-1*, conjugative transfer region II (C2, ~36 kb), and the *ter* operon conferring tellurite resistance (E). There were slight decrease in the regions between *repHI2* and IS*26*_1, including C1 (Fig. 1; Fig. S1; Data S2).

Exposure to CTX at high concentrations resulted in increases in gene relative abundance ranging from 165% to 250% for most genes from D2. In contrast, in the CTX-low and CTX-middle groups, gene abundance did not change and decreased to ~50% in D2, respectively, except for a 4.002 kb fragment containing truncated IS*26-fosA3-bla*_CTX-M-14_, where gene levels were retained at 107%–129%. The gene relative abundance sharply decreased to 20%–30% in the *intl1*-region from D1 in the CTX-middle group but was maintained at levels of 50%–80% for the CTX-high and CTX-low groups. The gene relative abundance of *oqxAB* from D1 was almost lost in all CTX exposure groups (Fig. 1B; Fig. S1; Data S2)

In our no-drug and combination treatment groups for the 15 endpoint-evolved populations, there were specific alterations. The gene average relative abundance in D2 was mostly retained at relatively high levels (75%–100%) for the CTX-CIP, CTX-CS, and CTX-CIP-CS groups. In contrast, this region was represented at levels of 25%–35% for the no-drug and CIP-CS groups. Moreover, the presence of *oqxAB* from D1 was almost lost in the no-drug and CTX-CS groups, whereas it was maintained at relatively low levels for CTX-CIP-CS (10%–15%), CIP-CS (20%–25%), and CTX-CIP (65%–75%). The *intl1*-region from D1 was detected at low levels (5%–10%) in the no-drug groups, whereas it was maintained at relatively low levels for the CTX-CIP-CS and CIP-CS groups (20%–30%), CTX-CIP (60%–70%), and CTX-CS (50%–80%). Furthermore, the CIP-CS group displayed sharp decreases to 36%–58% in the partial C1 region between *trhG* and IS*26_1*, but it slightly increased (100%–150%) in most genes in the regions between *dcm* and *insAB* (Fig. 1C; Fig. S1; Data S3).

Taken together, WGS analyses of 14028-pJXP9-evolved populations revealed that long-term antibiotic selections for pJXP9 (~600 generations) resulted in larger alterations of the plasmid compared to relatively short-term antibiotic selections (~280 generations). These alterations primarily occurred in the plasmid MDR regions. Specifically, ARGs from MDR regions, particularly *oqxAB* and the *intl1*-region, were largely lost in both CS and CIP treatment groups, especially at low concentrations. Similar scenarios were observed in MDR region I from the CTX groups, whereas gene amplifications were found in MDR region II from the CTX-high group. Exposure to single antibiotic treatment with different concentration treatments also changed the gene relative abundance in pJXP9 MDR regions from the endpoint-evolved populations, particularly in the CTX treatment group, but the changes seemed to be limited in the CIP treatment group. In the combination treatments, ARGs from the MDR regions could be maintained at certain levels in the CTX-CIP, CTX-CS, and CTX-CIP-CS groups, whereas they were lost at high levels in the CIP-CS and no-drug groups. Furthermore, conjugative transfer region I was also lost to some extent from CS, CIP, and CS-CIP groups.

### Large reduction in plasmid-mediated antibiotic resistance among endpoint-evolved clones

**Fig 2:**
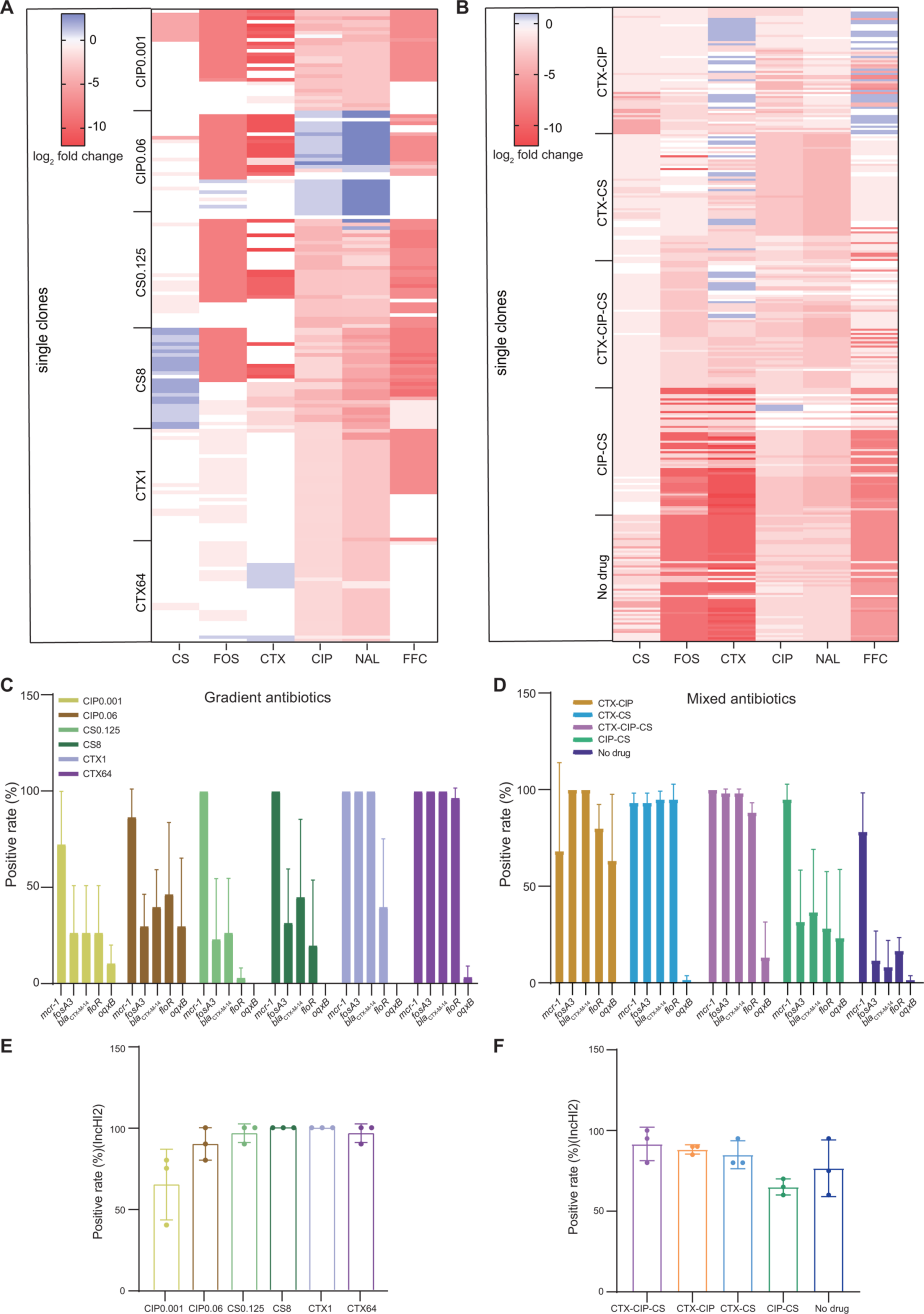
Change in plasmid-mediated antibiotic resistance and the plasmid maintenance among endpoint-evolved populations/clones. MIC changes for (**A**) 175 endpoint-evolved clones from mono-therapies and (**B**) 300 endpoint-evolved clones from combination-drug and no-drug treatments compared to the ancestral strain ATCC14028 carrying pJXP9. The positive detection rates of five pJXP9-encoded ARGs and *repHI2* among (**C and E**) 175 endpoint-evolved clones from mono-drug treatments and (**D and F**) 300 endpoint-evolved clones from combination-drug and no-drug treatments.

The data above indicate that the MDR regions of the pJXP9 plasmid were extensively altered among the endpoint-evolved populations. Therefore, we conducted additional phenotypic antibiotic susceptibility testing (MIC analysis) and PCR gene identification for these groups. Regarding single mono-drug treatments at high or low concentrations, the change in gene relative abundance in the pJXP9 MDR regions from the endpoint-evolved populations was higher than that at the middle concentration, and we chose 475 endpoint-evolved clones, including 175 from mono-drug treatments for CS, CTX, and CIP at low and high concentrations (8–10 clones from each independent population) and 300 from combinational drug and no-drug treatments (20 clones from each independent population). We investigated five pJXP9-encoded ARGs, which were clinically important ARGs, and they included *mcr-1*, *bla*_CTX-M-14_, *oqxAB*, *fosA3*, and *floR*. Similarly, these ARGs confer resistance to respectively corresponding antibiotics, and six corresponding antibiotics (including CS, CTX, CIP, NAL, FOS, and FFC) were chosen for drug susceptibility tests. Interestingly, the MICs for the six tested drugs mostly decreased in these populations compared to the ancestral strain ATCC14028 carrying pJXP9 (Fig. 2A and B; Data S4 and S5). Consistently, the majority of the five ARGs were lost to some extent (Fig. 2C and D; Data S6 and S7). In the mono-drug groups, the MICs dropped, and ARGs were lost to the largest extents from the CIP and CS groups. Particularly, low-level drug exposure groups led to larger MIC reductions and ARG losses compared to high-level exposure groups (Fig. 2A and C). In the combination and no-drug groups, the MICs and ARGs were lost to the largest extent in the no-drug group, and the other groups could be ranked as CIP-CS > CTX-CIP-CS > CTX-CS > CTX-CIP (Fig. 2B and D).

Notably, the MICs for ciprofloxacin and nalidixic acid decreased in most clones from all treatment groups except for the CIP-high group where they increased. The gene *oqxB* was almost lost from the CS, CTX, CTX-CS, and no-drug groups. Furthermore, a high-level reduction for *oqxB* was found even in the CIP mono and combination treatments. Moreover, the MICs for cefotaxime, fosfomycin, and florfenicol sharply dropped, and *bla*_CTX-M-14_, *fosA3*, and *floR* were largely lost among most of endpoint-evolved clones from the mono-CIP and CS groups as well as for CIP-CS and no-drug groups. In contrast, *fosA3*, *bla*_CTX-M-14_, and *floR* were maintained at high levels at the presence of the mono-drug CTX and CTX combination treatments. In a subset of the endpoint-evolved clones from the no-drug, CTX-CIP, and CIP groups, the MICs for CS decreased, and *mcr-1* was lost at low levels, whereas it was retained at high levels in other treatment groups (Fig. 2A through D). Furthermore, the MICs for CS were slightly increased in most endpoint-evolved clones in the CS-high groups. Additionally, a minority of clones displayed slight MIC increases for cefotaxime (53 clones) from CTX-CIP, CTX-CS, CTX-CIP-CS, and CTX-high groups, for florfenicol (27 clones) from CTX-CIP group, for ciprofloxacin (three clones) from CIP-CS group, and for fosfomycin (four clones) from CTX-high and CIP-high groups (Fig. 2A and B).

In addition to pJXP9-encoded ARGs, the plasmid stability of pJXP9 was also determined among the endpoint-evolved clones by examining the presence of *repHI2* gene. In the 175 endpoint-evolved clones from mono-drug treatments as described above, *repHI2* was detected in 90%–100% of the isolates from mono-drug CS and CTX treatments. The plasmid was present in 40%–80% and 80%–100% of clones in mono-drug treatments of CIP at low and high concentrations, respectively (Fig. 2E). Among 300 endpoint-evolved clones from combination and no-drug treatments as described above, the prevalence of *repHI2* was as follows for the groups was CTX-CIP-CS (80%–100%), CTX-CIP (85%–90%), CTX-CS (80%–95%), no-drug (60%–95%), and CIP-CS (60%–70%; Fig. 2F). Taken together, these results indicated that after co-culture of pJXP9 and strain 14028 for 63 days, plasmid pJXP9 could be maintained at high levels in most of 14028 populations from all treatment groups, even in the absence of drug. However, plasmid-mediated drug resistance encoded by the pJXP9 MDR region was generally lost in most endpoint-evolved clones from the no-drug, mono-CS, and mono-CIP groups, as well as the CIP-CS groups. We also found that *oqxB* was lost at high levels, even in the mono-CIP and CIP combination groups, in addition to its almost complete loss in other groups, including no-drug group. In contrast, plasmid-mediated drug resistance encoded by *bla*_CTX-M-14_, *fosA3*, and *floR* was generally retained in most of endpoint-evolved clones from the mono-CTX and CTX combination groups. Overall, *mcr-1* was generally retained at high levels from all groups, particularly for mono-CS and CS combination groups.

### Diverse deletions of the plasmid MDR region from endpoint-evolved clones under different positive selections

**Fig 3:**
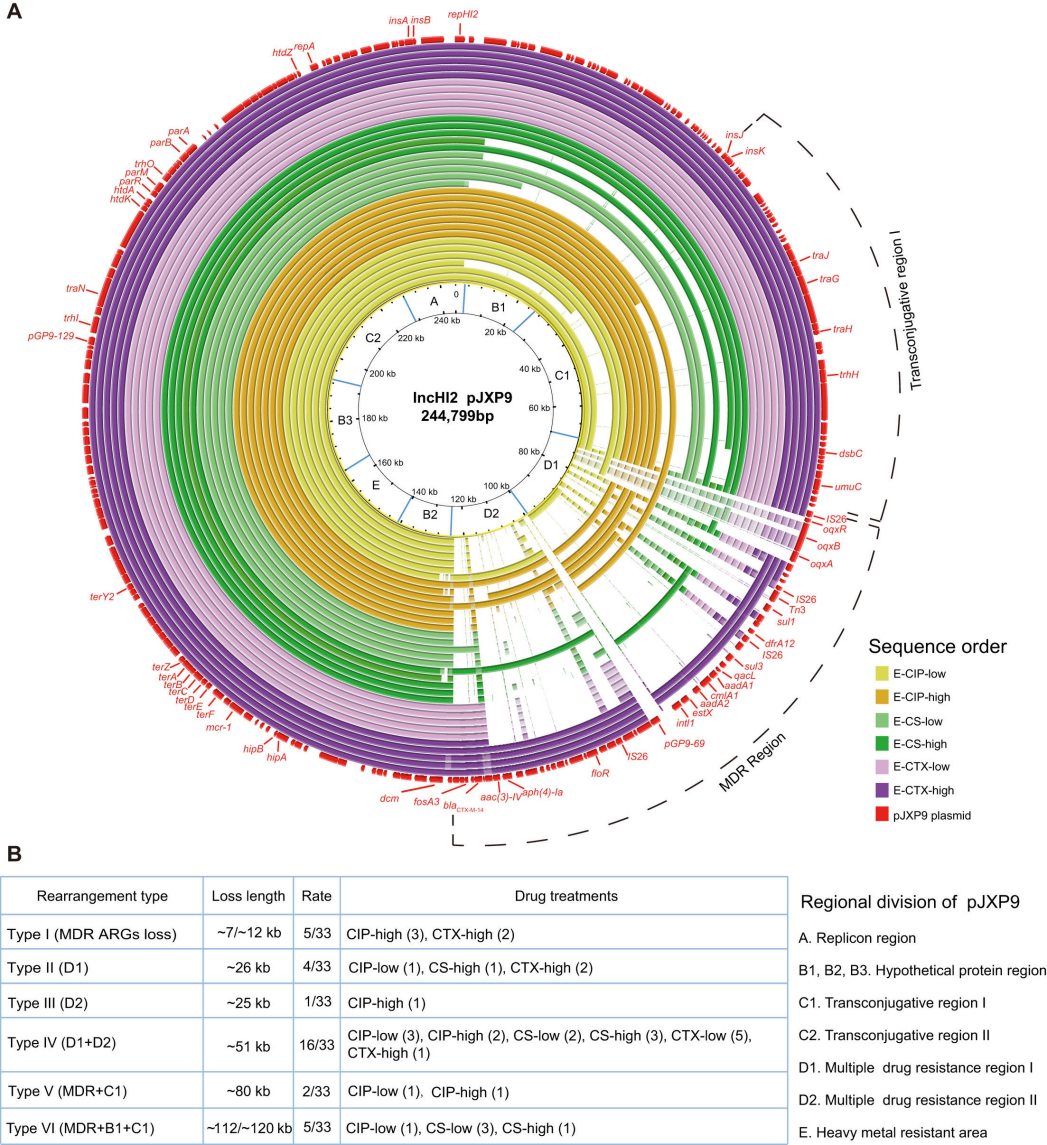
Sequence analysis of pJXP9 plasmids from endpoint-evolved clones from the CS, CTX, and CIP (low and high) mono-drug treatment groups. (**A**) Alignment of pJXP9 plasmids from 33 endpoint-evolved clones with the ancestral plasmid pJXP9 using BRIG. (**B**) Summary information of deletion profiles for the plasmid pJXP9 from 33 endpoint-evolved clones.

To further investigate the evolution of plasmid pJXP9 in *Salmonella* through positive selection, we obtained WGS data from 63 endpoint-evolved clones carrying pJXP9 plasmid. These clones comprised 33 clones from the mono-drug groups CS, CTX, and CIP at low and high concentrations (5–7 clones per group) and 30 clones from the combination and no-drug groups (six clones per group). Initially, we mapped the evolved plasmid pJXP9 and identified a total of six types of deletions ranging in size from 7 to 120 kb. The first type, Type I (MDR ARG loss) consisted of five clones with deletions of approximately 7–12 kb fragment containing ARGs from the MDR region. The other five deletion types involved the following plasmid regions: MDR region I (D1) with four clones (Type II); MDR region II (D2) with one clone (Type III); D1 and D2 with 16 clones (Type VI); MDR region and conjugative transfer region I (C1) with two clones (Type V); a region containing hypothetical coding sequences (B1), MDR region, and C1 with five clones (Type VI; Fig. 3A and B).

**Fig 4:**
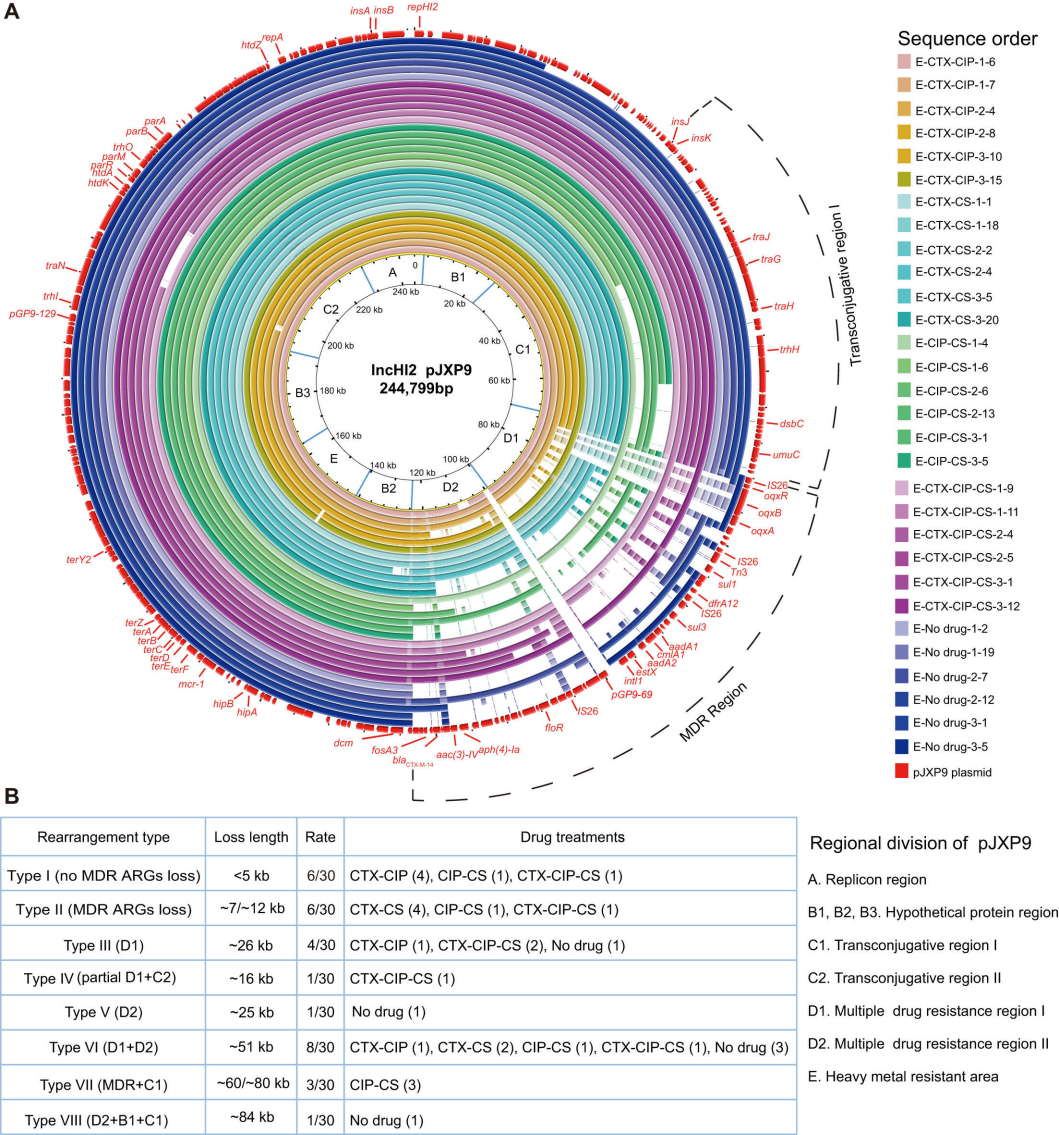
Sequence analysis of pJXP9 plasmids from endpoint-evolved clones from combination-drug and no-drug groups. (**A**) Alignment of pJXP9 plasmids from 30 endpoint-evolved clones with the ancestral plasmid pJXP9 using BRIG. (**B**) Summary information of deletion profiles for the plasmid pJXP9 from 30 endpoint-evolved clones.

Regarding the evolved plasmid pJXP9 from combination-drug and no-drug groups, a total of eight deletion types were observed, with deletion sizes ranging from less than 5 kb to approximately 84 kb (Fig. 4A and B). Type I (no MDR ARG loss) comprised six clones with deletions of the gene *pGP9-69* and/or *mcr-1*, each less than 5 kb in size. Type II (MDR ARGs loss) included six clones with deletions of approximately 7–12 kb fragments containing ARGs from the MDR region. The other six deletion types involved the following plasmid regions: D1 with four clones (Type III); D1 and C2 with one clone (Type IV); D2 with one clone (Type V); D1 and D2 with eight clones (Type VI); MDR region and C1 with three clones (Type VII); D2, C1, and B1 with one clone (Type VIII). The size of pJXP9 from the endpoint-evolved 14028-pJXP9 clones was confirmed by the gene location of *repHI2* among 12 selected evolved clones from the CTX-CIP and CTX-CS groups (Fig. 4A and B; Fig. S2).

Interestingly, the deletion type observed in evolved plasmid pJXP9 was related to the treatment conditions. In the CTX-high group, deletions were primarily distributed as Types I-II, each less than 30 kb in size. In the CTX-low and CS-high groups, Type VI deletions (~51 kb) were predominant. In the CS-low and CIP-low groups, deletions were mainly Types IV and VI (~51 kb and ~120 kb, respectively). In the no-drug and CIP-CS groups exhibited primarily Types VI and VIII deletions (~51 to ~84 kb), whereas CTX-CIP-CS, CTX-CIP, and CTX-CS groups displayed mainly Type I and III deletions, ranging from less than 5 kb to approximately 26 kb in size (Fig. 3B and 4B).

In summary, the WGS analyses of endpoint-evolved 14028-pJXP9 clones revealed diverse deletions in plasmid pJXP9, primarily occurring in the MDR region and, to a lesser extent, in conjugative transfer region I. In particular, we found large deletions in the overall MDR region in the no-drug, CS, CIP, and CTX-low groups, as well as CIP-CS groups. Additionally, deletions of conjugative transfer region I were found in clones exposed to mono-CS, mono-CIP, and CIP-CS conditions. However, MDR region deletions were less frequent in CTX-high group and CTX combination groups.

### Deletions of plasmid MDR region associated with improved growth and competitive advantages

**Fig 5:**
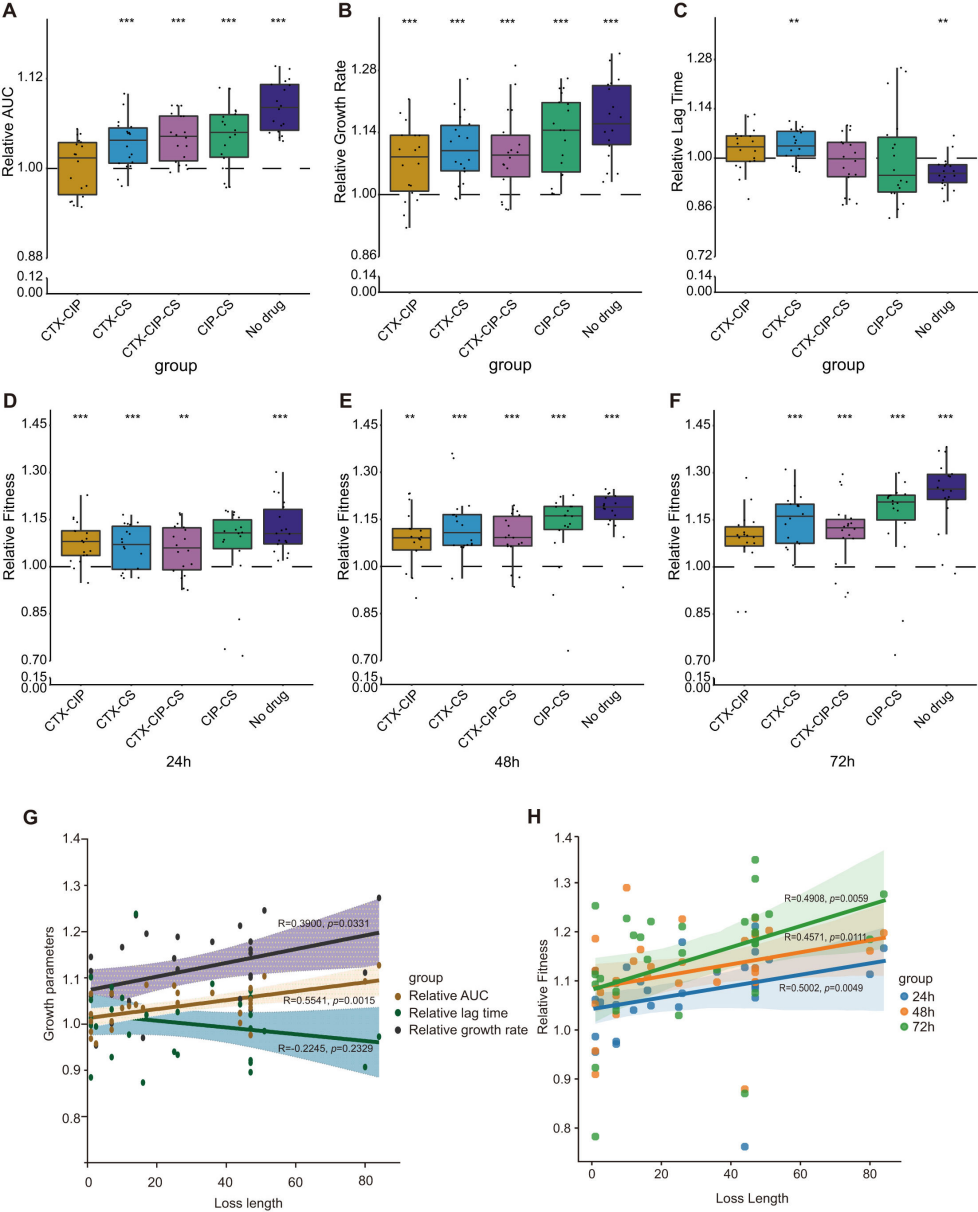
Growth kinetics and competition capability of 30 endpoint-evolved 14028-pJXP9 clones from combination-drug and no-drug groups relative to the ancestral 14028-pJXP9 strain. Relative (**A**) AUC, (**B**) growth rate and (**C**) lag time. Relative competition fitness at (**D**) 24, (**E**) 48, and (**F**) 72 h. Correlations between the length of deleted segments of plasmid pJXP9 from endpoint-evolved 14028-pJXP9 clones and (**G**) growth kinetics and (**H**) competition capability relative to pJXP9 from the ancestral 14028-pJXP9 strain. Statistical significance was calculated using paired sample *t*-test or nonparametric Mann–Whitney *U*-test. *, *P* < 0.05; **, *P* < 0.01; and ***, *P* < 0.001.

To determine the fitness response to plasmid-bacterial host coculture, with or without antibiotic treatments, we utilized the ancestral 14028-pJXP9 and 30 endpoint-evolved 14028-pJXP9 clones obtained from combination-drug and no-drug exposures, as described above in WGS analysis of endpoint-evolved clones. We examined growth kinetics and competition capabilities for these groups and observed growth fitness advantages in almost all 30 endpoint-evolved 14028-pJXP9 clones after a 12-h assessment (Fig. S3). The relative AUCs and maximum growth rates were significantly increased for all 30 endpoint-evolved 14028-pJXP9 clones compared to the ancestral 14028-pJXP9 strain. The highest relative fitness advantages could be ranked as follows: no-drug > CIP-CS > CTX-CIP-CS > CTX-CS > CTX-CIP (Fig. 5A and B). In contrast, the relative lag times were largely reduced in the endpoint-evolved 14028-pJXP9 clones from the no-drug (*P* < 0.01) and CIP-CS (*P* > 0.05) groups, while slightly increased in the CTX-CS (*P* < 0.01) and CTX-CIP (*P* > 0.05) groups. This parameter remained stable for the CTX-CIP-CS group (Fig. 5C). Direct competition assays revealed that almost all 30 endpoint-evolved 14028-pJXP9 clones significantly outnumbered the ancestral 14028-pJXP9 strain after 24, 48, and 72 h of cultivation. The competitive advantage was also slightly increased with each serial passage. In particular, the highest competitive advantage in the 14028-pJXP9 evolved clones could be ranked as no-drug and CIP-CS groups, CTX-CS, and CTX-CIP-CS groups and then the CTX-CIP group (Fig. 5D through F).

**Fig 6:**
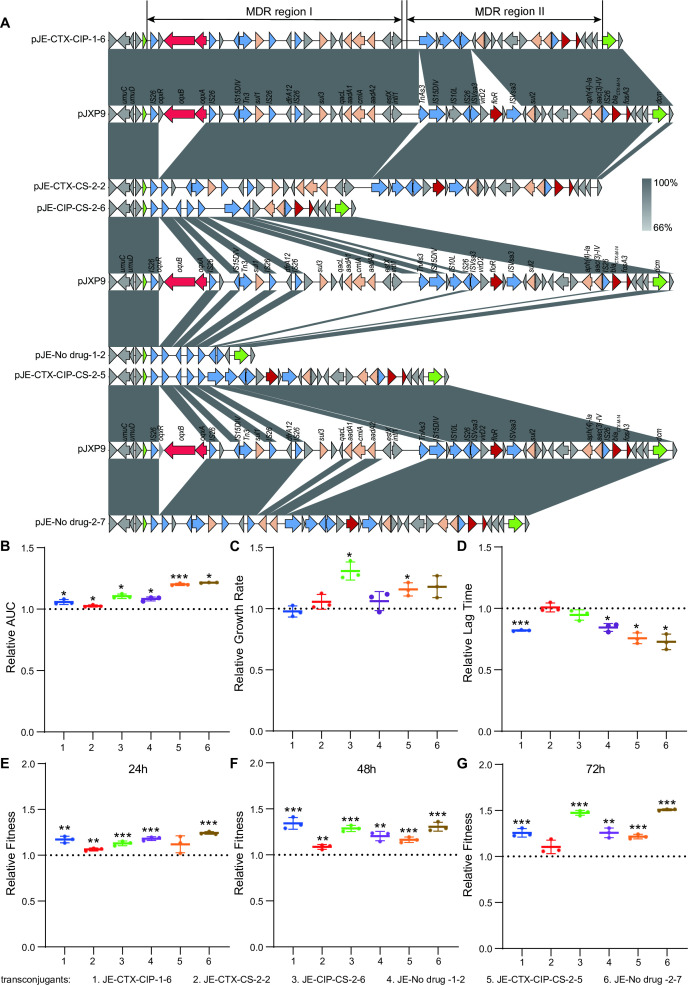
Sequence analysis of MDR region from six 14028-rif-evolved pJXP9 transconjugants, and the effect of MDR region deletions on bacterial fitness cost. (**A**) The supposed genetic contexts of plasmid-borne MDR in six transconjugants bearing the evolved plasmid pJXP9 with Type II deletions (MDR ARGs loss), Type III deletions (MDR region I), and Type VI deletions (MDR region). Relative values for the (**B**) AUC, (**C**) growth rate and (**D**) lag time for six 14028-rif-evolved-pJXP9 transconjugants compared to the 14028-rif-pJXP9. Relative competitive fitness at (**E**) 24, (**F**) 48, and (**G**) 72 h. Statistical significance was calculated using paired sample *t*-test or nonparametric Mann–Whitney *U*-test. *, *P* < 0.05; **, *P* < 0.01; and ***, *P* < 0.001.

To determine the fitness effect of sequence fragment deletions from the evolved plasmid pJXP9, we correlated the relative fitness of endpoint-evolved 14028-pJXP9 clones relative to ancestral 14028-pJXP9 strain and the loss length of evolved plasmid pJXP9. Both the relative AUC (*R* = 0.5541, *P* < 0.01) and growth rate (*R* = 0.3900, *P* < 0.05) displayed a positive and statistically significant correlation with deletion length, while the relative lag time was not significantly related to deletion length (*R* = −0.2245, *P* > 0.05; Fig. 5G). Furthermore, the relative competition fitness at 72 h and the loss length remained significantly and positively correlated (*R* = 0.4908, *P* < 0.01; Fig. 5H). To further determine whether plasmid modifications, particularly MDR region deletions, contributed to alleviating the fitness cost of pJXP9 carriage, we transferred pJXP9 from endpoint-evolved 14028-pJXP9 clones that displayed reduced plasmid-mediated antibiotic resistance (lowered MIC) to rifampicin-resistant *S*. Typhimurium ATCC14028 (14028-rif). We obtained six transconjugants carrying evolved pJXP9 (14028-rif-evolved-pJXP9 transconjugants) with three deletion types, as judged by both MIC tests and sequence analysis. These included Type II (MDR ARGs loss; *n* = 2), Type III (D1, MDR region I; *n* = 2), and Type VI (D1 and D2, MDR region; *n* = 2; Fig. 6A and Fig. 4B; Table S3). Overall, the growth fitness advantage was increased for these six transconjugants when compared to the 14028-rif ancestral strain carrying pJXP9 (14028-rif-pJXP9) after a 12-h assessment (Fig. 6B through D). Similarly, six 14028-rif-evolved pJXP9 transconjugants significantly outnumbered the 14028-rif-pJXP9 after 24, 48, and 72 h of co-cultivation (Fig. 6E through G). Moreover, on the whole, the highest growth and competition advantages were observed in transconjugants carrying evolved pJXP9 with Type III deletions.

Taken together, these results revealed that after co-culture of pJXP9 and 14028 for 63 days, both the growth and competition advantages were improved among endpoint-evolved 14028-pJXP9 clones from all combination-drug and no-drug groups, particularly for the no-drug and CIP-CS groups. Moreover, the increase in deletion length of plasmid pJXP9 was associated with improved fitness for the endpoint-evolved 14028-pJXP9 clones. Furthermore, the deletion of plasmid pJXP9 MDR region and especially MDR Region I could mitigate the fitness cost inflicted by pJXP9 plasmid carriage.

### Parallel mutations in a chromosome-encoded *arcA* gene associated with carriage of pJXP9 plasmid among endpoint-evolved clones

**Fig 7:**
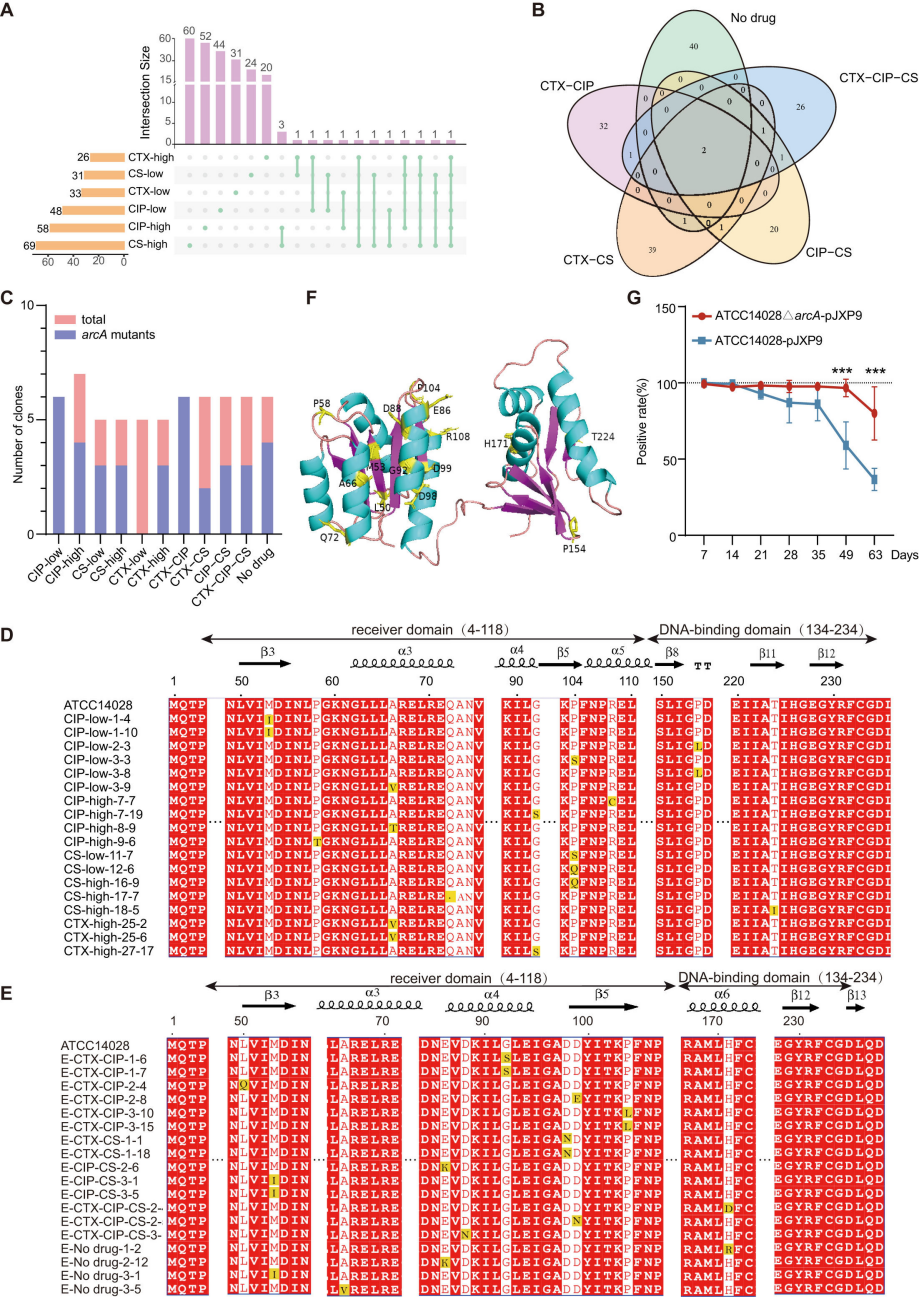
Chromosomal gene mutations analysis among endpoint-evolved clones. Shared and unique mutated genes among endpoint-evolved clones from (**A**) mono-drug groups of CS, CTX, and CIP with low and high concentrations and from (**B**) combination-drug and no-drug groups. (**C**) The number of clones possessing *arcA* mutation in each of treatment groups. ArcA amino acid mutation sites among 18 endpoint-evolved 14028-pJXP9 clones from mono-drug groups (**D**) and 18 ones from combination-drug and no-drug groups (**E**). (**F**) Summary of all amino acid substitutions in *arcA* from 36 endpoint-evolved 14028-pJXP9 clones from both mono-drug and combination-drug, as well as no-drug groups. (**G**) The pJXP9 plasmid stability effects of *arcA* deletion in 14028∆*arcA*-pJXP9 and 14028-pJXP9. Statistical significance was calculated using Chi-squared test. ***, *P* < 0.001.

To determine whether chromosomal modifications were associated with carriage of pJXP9 plasmid, we analyzed the chromosomal gene mutations from endpoint-evolved clones. Total 245 and 164 chromosomal mutation genes associated with pJXP9 plasmid carriage were identified from mono-drug groups (33 endpoint-evolved clones) and from combination-drug and no-drug groups (30 endpoint-evolved clones), respectively, and these excluded mutated genes found in the ancestral 14028 evolved clones (Fig. 7A and B; Data S8 and S9). Of note, endpoint-evolved 14028-pJXP9 clones from different treatments possessed almost fully different mutated chromosomal genes. Only one mutation gene, *arcA* (a global regulator), was found among endpoint-evolved clones from all mono-drug groups except for CTX-low group, and only two genes, *arcA* and *manA* (encoding mannose-6-phosphate isomerase), were found among endpoint-evolved clones from all treatments. Overall, *arcA* mutation was found among almost all of treatment groups but CTX-low group, and it was found at the highest frequencies in endpoint-evolved 14028-pJXP9 clones from CIP-low and CTX-CIP groups (Fig. 7C). Moreover, mutation in *arcA* was identified at the highest frequencies among 36 of 63 (57.1%) endpoint-evolved 14028-pJXP9 clones, each possessed a single amino-acid change (Fig. 7D and E), and 20 amino acid substitutions were found. Amino acid substitutions M53I (*n* = 5), V66A (*n* = 4)/T66A (*n* = 1), S92G (*n* = 4), and S104P (*n* = 2)/Q104P (*n* = 2)/L104P (*n* = 2) were found at relatively high frequencies, and they occurred at both mono-drug and combination-drug/no-drug groups (Table S4; Fig. 7F). In addition, amino acid substitutions were identified in the receiver domain and DNA-binding domain of ArcA among 31 and 5 endpoint-evolved 14028-pJXP9 clones, respectively (Fig. 7F).

Given diverse amino acid substitutions identified in *arcA*, we therefore generated *arcA* null mutants in the ancestral strains 14028 (14028∆*arcA*) and the ancestral 14028-pJXP9 (14028∆*arcA*-pJXP9) to determine whether *arcA* mutations were involved in adaptive evolution between pJXP9 and 14028 diverse positive selections. Initially, we assessed the fitness cost of plasmid carriage in the presence or deletion of *arcA*. We found that *arcA* deletion led to a slightly reduced growth and competitive advantages in both the ancestral 14028 and ancestral 14028-pJXP9 (Fig. S4). However, *arcA* deletion improved the stability of pJXP9 in 14028 from day 21, in particular at 49 and 63 days (*P* < 0.05) in an antibiotic-free environment (Fig. 7G).

Taken together, parallel mutations in chromosomal *arcA* were found to be associated with pJXP9 plasmid carriage among endpoint-evolved clones from diverse treatments. Meanwhile, *arcA* deletion improved the persistence of pJXP9 plasmid without the selective pressure of antibiotics.

## DISCUSSION

Plasmid-mediated ARG dissemination has become a great concern to human health and especially for MDR plasmids that both cause the rapid spread of MDR bacteria and complicate clinical treatment options. However, we still have a limited understanding of the dynamics of plasmid-mediated antibiotic resistance and the evolutionary adaptation of MDR plasmid-bacteria pairs under different antibiotic selection pressures. To address this, we conducted a study to investigate the co-adaptation between the MDR IncHI2 plasmid pJXP9 and *S. enterica* serovar Typhimurium strain ATCC 14028 under diverse antibiotic selection conditions. Our goal was to gain insights into the evolutionary changes that occurred in plasmids and plasmid-mediated antibiotic resistance for co-adaptation of MDR plasmid-bacteria pairs under different antibiotic selection pressures.

During our study, we observed that the maintenance of plasmid-mediated antibiotic resistance varied under different drug selection conditions. Generally, plasmid-encoded ARGs were maintained under selection for the corresponding antibiotic. For instance, the *bla*_CTX-M-14_ gene was consistently maintained in most endpoint-evolved clones/populations from CTX and CTX combination treatments. Similarly, the *oqxB* gene was predominantly lost in all endpoint-evolved clones/populations, except for those exposed to mono-CIP and combinational CIP treatments where it was maintained at higher levels. These findings align with the concept that exposure to antibiotics can enrich and maintain amplified resistance genes located on both the plasmid and the chromosome (33). Furthermore, *fosA3* and *floR* were physically adjacent to *bla*_CTX-M-14_ and were retained at high levels similar to *bla*_CTX-M-14_ among most of endpoint-evolved clones from the mono-CTX and CTX combination treatments. Unexpectedly, this was not the case for *oqxB* which was physically most distant from *bla*_CTX-M-14_ even though all these four ARGs were located on a single plasmid-encoded MDR region. These findings emphasize that closely linked ARGs can be co-selected, while ARGs located farther apart might experience weaker co-selection. Besides the co-resistance that ARGs present on a single mobile genetic element, such as plasmids, co-regulation also was recognized as a mechanism of co-selection (34). Further study is required to determine the regulatory mechanism involved in plasmid-encoded ARGs in MDR region under the corresponding antibiotic treatments, which might help to reveal the relationship between the co-selection of ARGs and their genetic distance on a single plasmid.

More importantly, we found that changes in plasmid pJXP9 primarily occurred in the MDR regions and, to a lesser extent, in the conjugative transfer region I among both endpoint-evolved 14028-pJXP9 populations and individual clones. These alterations were often in the form of large deletions. Interestingly, the extent of alterations correlated with the duration of exposure to a particular antibiotic since we measured the relative abundance of genes and observed more significant alterations in endpoint-evolved populations (transfer day 63) compared to midpoint-evolved populations (transfer day 28). Importantly, different drug selection conditions led to distinct alterations in the MDR regions. For example, exposure to CTX resulted in the loss of MDR region I that contained *oqxAB*, while ARGs copies in MDR region II (containing *bla*_CTX-M-14_, *fosA3*, and *floR*) were amplified in the high-CTX group. Additionally, combinational exposures of CTX led to the maintenance of overall MDR region. On the other hand, exposure to CS or CIP, either alone or in combination, promoted the loss of MDR region and conjugative transfer region I.

Exposure to a specific antibiotic could promote the expression of corresponding ARG and also amplify the number of corresponding ARG copies (33, 35). The gene expression or the gene copy number of *oqxAB*, *mcr-1*, and *bla*_CTX-M-14_ could possibly be enhanced under the sub-MIC ciprofloxacin, colistin, and cefotaxime, respectively. It is notable that the acquisition or increased expression of *oqxAB* and *mcr-1* was detrimental and posed large fitness costs to bacteria (36–38). By contrast, inactivation of *bla*_CTX-M-14_ in plasmid pCT had no effect on the fitness cost to its host bacteria (38, 39). Therefore, we speculated that exposure to colistin or ciprofloxacin promoted the loss of MDR region and conjugative transfer region, resulting in relieving the fitness costs that possibly caused by the increased expression or the number of gene copies of *oqxAB* or *mcr-1*. Furthermore, it is also required to determine the regulatory mechanism involved in fragment deletion and homologous recombination occurring on plasmid, in particular MDR regions, with or without antibiotic treatments.

In addition to plasmid modification, strong parallel mutations in chromosomal *arcA* were observed and were associated with pJXP9 carriage among endpoint-evolved clones from diverse treatments. More importantly, an *arcA* null strain displayed elevated pJXP9 maintenance in 14028. Similarly, *arcA* mutations have been identified that facilitated MDR plasmid maintenance in clinical *E. coli* strains, although this was found during bacterial niche adaptation and independently of plasmid carriage (7). ArcA is a global transcriptional regulator that regulates central metabolic and redox homeostatic pathways (40). Since carriage of MDR plasmids is frequently accompanied by energy costs and metabolic loads, *arcA* inactivating mutations might result in metabolic reprogramming and accelerating loss of plasmid-encoded MDR region that in turn facilitates plasmid maintenance. It is still required to explore how *arcA* inactivating mutations were related to MDR plasmid maintenance.

Our findings of drug resistance and bacterial fitness phenotypes in endpoint-evolved 14028-pJXP9 clones from combination-drug and no-drug groups suggested that adaptive evolution of 14028-pJXP9 pair is a trade-off between plasmid-mediated antimicrobial resistance (AMR) and the fitness cost of plasmid carriage. Furthermore, fitness-resistance trade-offs were most likely due to pJXP9 plasmid modifications and, in particular, MDR region deletions. Similar scenarios were also observed in ours and other studies using *in vitro* experimentally evolved *S*. Typhimurium, *Klebsiella pneumoniae*, and *Staphylococcus aureus* carrying MDR plasmids in the presence or absence of antibiotics (5, 6, 19) as well as *in vivo-*evolved OXA-plasmid-mediated ARG dissemination (41). These plasmids not only contribute to the rapid spread of MDR bacteria but also complicate clinical treatment options. Previous studies have indicated that the costs associated with AMR plasmids primarily arise from ARG expression (1, 42). Therefore, plasmid modifications are likely to result in the loss of antibiotic resistance, particularly when cultured without antibiotics, and thus reduce the fitness costs associated with plasmid carriage. If this trade-off is clinically relevant, reducing antibiotic use could lead to the loss of resistance encoded by MDR plasmids. However, of note, the re-establishment of successful plasmid-bacteria combinations could result in the re-acquisition of resistance in environments contaminated with ARGs and antibiotics. These indicated that it is complex to fight antibiotic resistance in bacteria, and the reduction and rational use of antimicrobials are essential.

In summary, our study provides insights into the co-adaptation of MDR plasmids and bacteria under different antibiotic selection pressures. We observed diverse genetic alterations in plasmids particularly in the MDR regions and parallel mutations in chromosomal *arcA* from diverse treatments. Additionally, the maintenance of plasmid-mediated antibiotic resistance varied depending on the selection pressure, and co-selection of ARGs depended on gene genetic distance. *arcA* deletion improved the persistence of pJXP9 plasmid without drugs. These findings contribute to our understanding of plasmid-mediated ARG dissemination and highlight the need for further research to unravel the mechanisms underlying the evolution of plasmid-borne MDR regions and the trade-offs between resistance and fitness.

## Data Availability

All WGS data have been deposited in the GenBank database under accession numbers SRX23995306 to SRX2395359 and SRX24382906 to SRX24382957 (BioProject PRJNA1088097; https://www.ncbi.nlm.nih.gov/sra?linkname=bioproject_sra_all&from_uid=1088097).

## SUPPLEMENTAL MATERIAL

Supplemental Figures and Tables msystems.01197-24-s0001.docx Figure S1-S4 and Tables S1-S4.

Supplemental Material msystems.01197-24-s0002.xlsx Data S1-S9.

ASM does not own the copyrights to Supplemental Material that may be linked to, or accessed through, an article. The authors have granted ASM a non-exclusive, world-wide license to publish the Supplemental Material files. Please contact the corresponding author directly for reuse.
