# Supplementary material for: Evolution and maintenance of a large multidrug-resistant plasmid in a Salmonella enterica Typhimurium host under differing antibiotic selection pressures
Source: mSystems. 2024 Oct 22;9(11):e01197-24. doi: 10.1128/msystems.01197-24 (PMC11575406; doi:10.1128/msystems.01197-24)
Supplement: Supplemental Figures and Tables — Figure S1-S4 and Tables S1-S4. [file msystems.01197-24-s0001.docx]

Supplementary materials for

**Evolution and maintenance of a large multidrug-resistant plasmid in a *Salmonella enterica* Typhimurium host under differing antibiotic selection pressures**

Ming Cheng^#1,2^, Jing-Jing Dai^#1,2^, Jing-Fei Zhang^1,2^, Yu-Ting Su^1,2^, Si-qi Guo^1,2^, Ruan-Yang Sun^1,2^, Dong Wang ^1,2^, Jian Sun^1,2^, Xiao-Ping Liao^1,2^, Sheng Chen^3*^, Liang-Xing Fang^1,2*^

^1^Guangdong Laboratory for Lingnan Modern Agriculture, National Risk Assessment Laboratory for Antimicrobial Resistance of Animal Original Bacteria, College of Veterinary Medicine, South China Agricultural University, Guangzhou, Guangdong, P. R. China

^2^Guangdong Provincial Key Laboratory of Veterinary Pharmaceutics Development and Safety Evaluation, South China Agricultural University, Guangzhou, Guangdong, P. R. China

^3^Department of Food Science and Nutrition, Faculty of Science, The Hong Kong Polytechnic University, Kowloon, Hong Kong SAR, P. R. China

*Corresponding author. E-mail: Liang-Xing Fang, fanglx@scau.edu.cn; Sheng Chen, Email: sheng.chen@polyu.edu.hk.

^#^These authors contributed equally to this work.


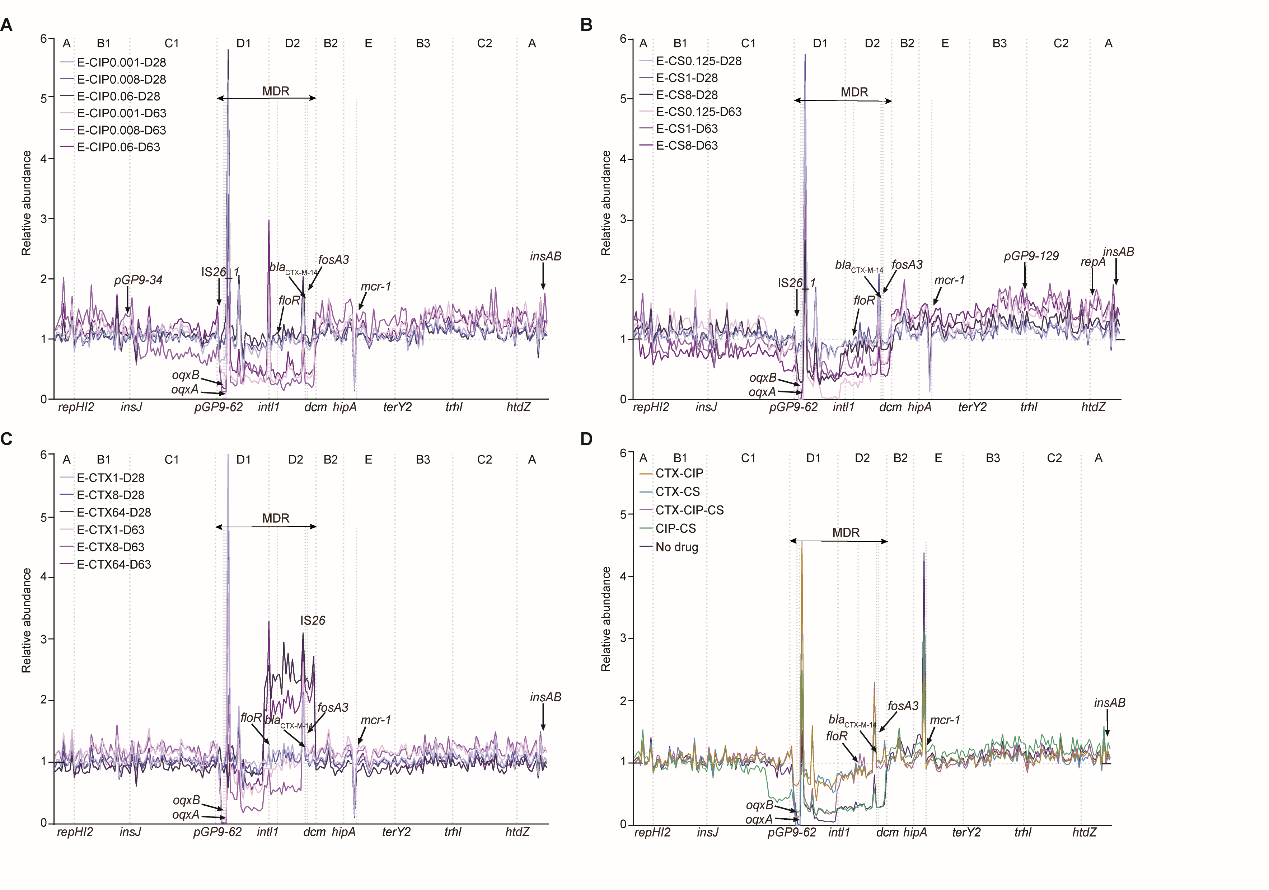


**Figure S1.** Relative abundance of genes in plasmid pJXP9 from mid-point and endpoint evolved populations for the indicated treatment groups. The gene relative abundance in pJXP9 from 9 mid-point and 9 endpoint evolved populations (one population of each treatment group) from mono-therapies using (A) CIP, (B) CS and (C) CTX with low, middle and high concentrations. (D) The gene average relative abundance in plasmid pJXP9 from 15 endpoint evolved populations (three populations of each treatment group) from No-drug group and combinations of CS, CTX and CIP groups.

**
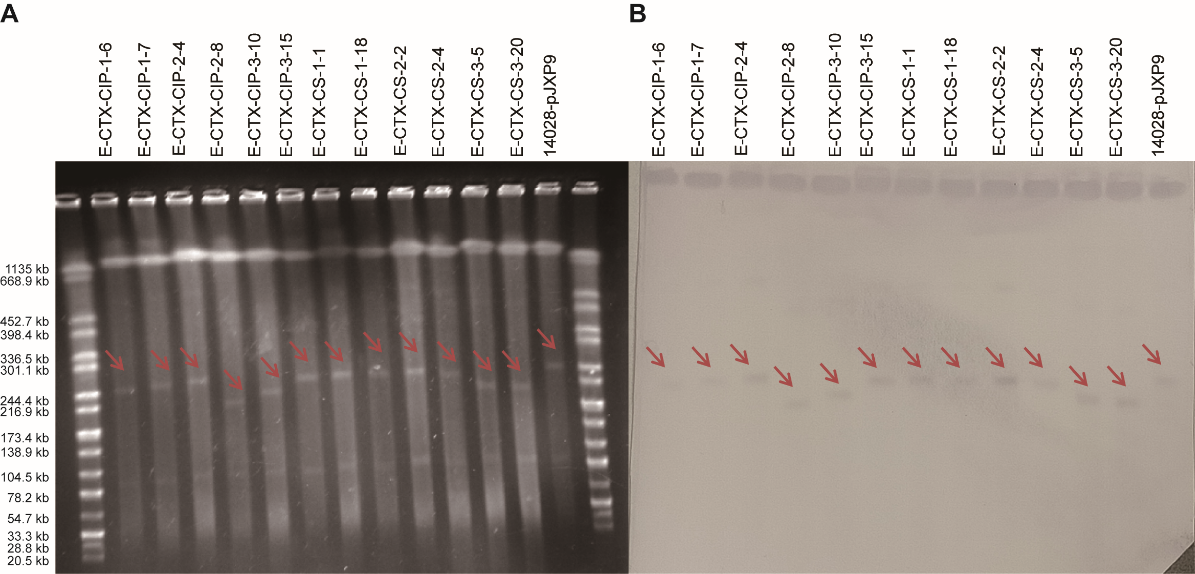
**

**Figure S2** Gene location of *repHI2* by (A) S1-PFGE and (B) hybridization analysis among the ancestral strain 14028-pJXP9 and 12 endpoint evolved clones bearing evolved plasmid pJXP9 from CTX-CIP and CTX-CS groups.

**
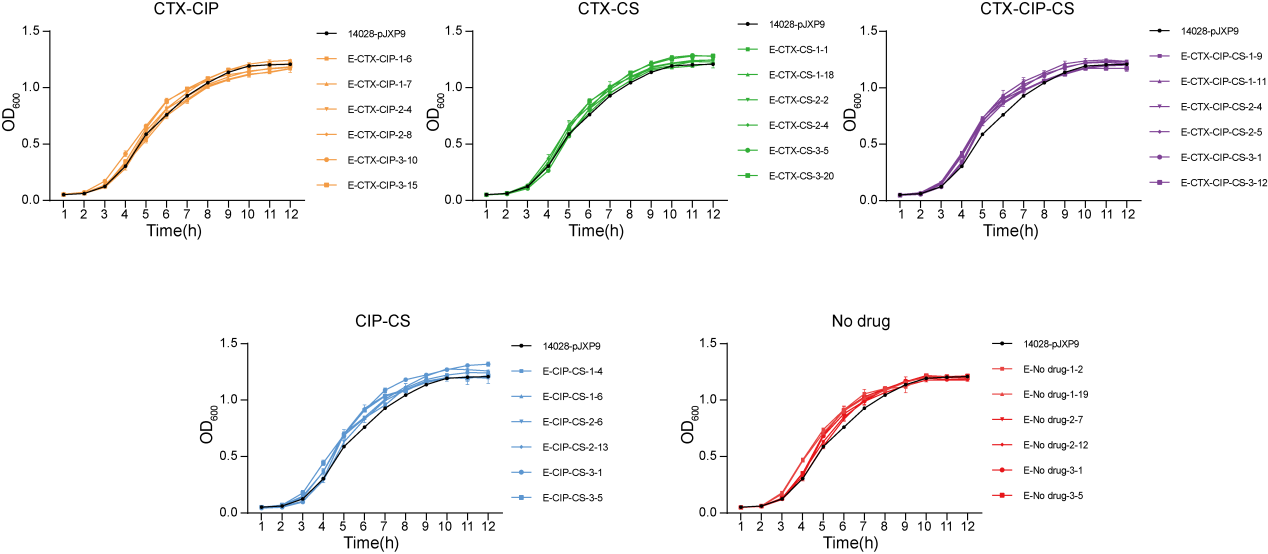
**

**Figure S3** Growth curves for the ancestral clone 14028-pJXP9 and 30 endpoint evolved clones bearing evolved plasmid pJXP9 from the indicated treatment groups.


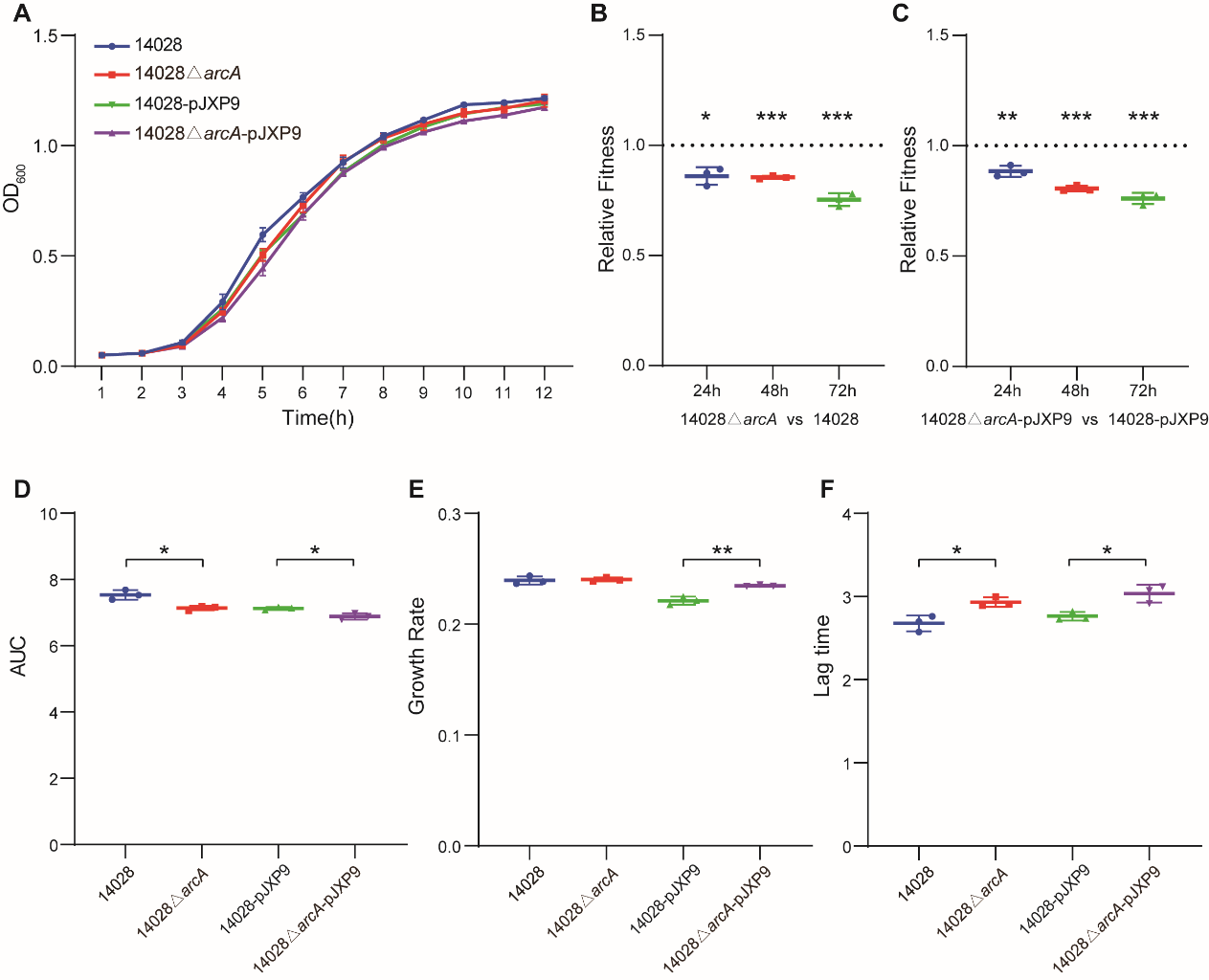


**Figure S4.** Growth and competition fitness cost effects of *arcA* deletion in both the ancestral 14028 and ancestral 14028-pJXP9 strains. (A) Growth curve of ancestral strain 14028 and ancestral 14028-pJXP9, with and without *arcA* deletion. (B) Relative competition fitness of *arcA* deletion in 14028 versus 14028 and (C) *arcA* deletion in 14028-pJXP9 versus 14028-pJXP9. (D-E) Growth AUC, rate and lag time of ancestral strain 14028 and ancestral 14028-pJXP9 with and without *arcA* deletion.

**Table S1.** Characteristics of sequencing quality and statistics per isolates

| Evolved clones | Assembled contigs | Largest contig | Total length | GC (%) | N50 | L50 | N's per 100 kbp | Average Coverage |
| --- | --- | --- | --- | --- | --- | --- | --- | --- |
| G1-4 | 57 | 622466 | 5075058 | 51.87 | 442050 | 5 | 1.83 | 254 |
| G1-10 | 57 | 622466 | 5059001 | 51.88 | 225811 | 6 | 1.92 | 287 |
| G2-3 | 57 | 622466 | 5020536 | 51.98 | 377311 | 5 | 3.86 | 232 |
| G3-3 | 60 | 522489 | 5023070 | 51.92 | 181370 | 8 | 1.93 | 244 |
| G3-8 | 57 | 622466 | 5094525 | 51.90 | 341636 | 6 | 1.88 | 276 |
| G3-9 | 61 | 622466 | 5114449 | 51.89 | 255064 | 6 | 1.82 | 292 |
| G7-6 | 69 | 622466 | 5133874 | 51.89 | 341636 | 6 | 3.80 | 221 |
| G7-7 | 53 | 622466 | 5087711 | 51.89 | 341636 | 6 | 3.79 | 236 |
| G7-19 | 66 | 622466 | 5121021 | 51.89 | 341636 | 6 | 3.77 | 237 |
| G8-3 | 71 | 622466 | 5134969 | 51.88 | 341636 | 6 | 1.89 | 194 |
| G8-9 | 57 | 622466 | 5107684 | 51.87 | 341636 | 6 | 1.82 | 255 |
| G9-3 | 55 | 622466 | 5056708 | 51.94 | 341248 | 6 | 1.84 | 207 |
| G9-6 | 64 | 622466 | 5112157 | 51.89 | 341635 | 6 | 1.82 | 251 |
| G10-1 | 54 | 662052 | 5019592 | 51.98 | 377311 | 5 | 1.85 | 286 |
| G10-5 | 56 | 622466 | 5027615 | 51.97 | 377311 | 5 | 1.93 | 246 |
| G11-7 | 57 | 622466 | 5071027 | 51.88 | 313635 | 6 | 1.89 | 281 |
| G11-16 | 60 | 622466 | 5077119 | 51.87 | 322384 | 6 | 1.93 | 216 |
| G12-6 | 55 | 622466 | 4981933 | 51.98 | 377311 | 5 | 1.87 | 318 |
| G16-4 | 61 | 536725 | 5075014 | 51.91 | 341636 | 6 | 3.8 | 263 |
| G16-19 | 58 | 622466 | 4958680 | 51.98 | 233136 | 6 | 1.88 | 223 |
| G17-1 | 64 | 587569 | 5086787 | 51.89 | 209048 | 8 | 11.4 | 235 |
| G17-7 | 60 | 438275 | 5085737 | 51.90 | 186766 | 10 | 0.00 | 320 |
| G18-5 | 352 | 622466 | 5283886 | 52.35 | 341636 | 6 | 3.03 | 244 |
| G19-2 | 59 | 622466 | 5101854 | 51.88 | 341636 | 6 | 1.82 | 198 |
| G19-3 | 58 | 622466 | 5101314 | 51.88 | 341636 | 6 | 1.88 | 280 |
| G20-1 | 59 | 622466 | 5099590 | 51.88 | 341636 | 6 | 5.75 | 259 |
| G21-6 | 59 | 622466 | 5099734 | 51.88 | 341636 | 6 | 1.88 | 319 |
| G21-8 | 58 | 622466 | 5101793 | 51.88 | 341636 | 6 | 1.90 | 226 |
| G25-2 | 61 | 622466 | 5099663 | 51.88 | 341636 | 6 | 1.90 | 234 |
| G25-6 | 61 | 622466 | 5116245 | 51.89 | 341636 | 6 | 1.88 | 274 |
| G26-5 | 70 | 622466 | 5135105 | 51.88 | 341636 | 6 | 1.91 | 233 |
| G27-6 | 71 | 622466 | 5134506 | 51.89 | 341636 | 6 | 3.74 | 227 |
| G27-17 | 63 | 622466 | 5117663 | 51.89 | 341636 | 6 | 1.82 | 199 |
| 1. CTX-CIP-1-6 | 85 | 622466 | 5141560 | 51.87 | 139247 | 10 | 1.89 | 271 |
| E-CTX-CIP-1-7 | 87 | 536598 | 5143686 | 51.89 | 156294 | 8 | 1.94 | 280 |
| E-CTX-CIP-2-4 | 91 | 539253 | 5118168 | 51.89 | 148498 | 10 | 1.95 | 307 |
| E-CTX-CIP-2-8 | 80 | 622466 | 5097434 | 51.87 | 156294 | 8 | 1.96 | 295 |
| E-CTX-CIP-3-10 | 83 | 622466 | 5117063 | 51.88 | 148500 | 10 | 0.00 | 241 |
| E-CTX-CIP-3-15 | 88 | 622466 | 5141092 | 51.88 | 156294 | 9 | 1.95 | 269 |
| E-CTX-CS-1-1 | 89 | 402844 | 5134734 | 51.88 | 150065 | 10 | 0.00 | 236 |
| E-CTX-CS-1-18 | 88 | 622466 | 5103341 | 51.87 | 165202 | 8 | 0.00 | 234 |
| E-CTX-CS-2-2 | 85 | 622466 | 5134158 | 51.88 | 209049 | 8 | 0.00 | 250 |
| E-CTX-CS-2-4 | 81 | 444617 | 5059187 | 51.90 | 140671 | 11 | 0.00 | 228 |
| E-CTX-CS-3-5 | 83 | 619154 | 5074704 | 51.87 | 150065 | 9 | 0.00 | 267 |
| E-CTX-CS-3-20 | 84 | 576240 | 5055234 | 51.87 | 156005 | 8 | 0.00 | 262 |
| E-CIP-CS-1-4 | 91 | 550738 | 5143888 | 51.88 | 143189 | 10 | 1.94 | 299 |
| E-CIP-CS-1-6 | 81 | 602665 | 5064812 | 51.92 | 148521 | 9 | 0.00 | 270 |
| E-CIP-CS-2-6 | 83 | 435129 | 5074643 | 51.87 | 142813 | 10 | 1.97 | 298 |
| E-CIP-CS-2-13 | 88 | 622466 | 5097656 | 51.86 | 139247 | 10 | 0.00 | 261 |
| E-CIP-CS-3-1 | 75 | 552671 | 5058373 | 51.89 | 149941 | 9 | 0.00 | 226 |
| E-CIP-CS-3-5 | 76 | 434952 | 5078838 | 51.89 | 156005 | 9 | 0.00 | 283 |
| E-CTX-CIP-CS-1-9 | 88 | 544685 | 5138736 | 51.87 | 149941 | 9 | 1.95 | 276 |
| E-CTX-CIP-CS-1-11 | 91 | 409929 | 5128093 | 51.90 | 149941 | 10 | 0.00 | 267 |
| E-CTX-CIP-CS-2-4 | 79 | 622460 | 5080611 | 51.87 | 209049 | 7 | 1.97 | 305 |
| E-CTX-CIP-CS-2-5 | 73 | 622466 | 5079740 | 51.91 | 149900 | 9 | 0.00 | 270 |
| E-CTX-CIP-CS-3-1 | 86 | 452427 | 5104616 | 51.88 | 150106 | 11 | 0.00 | 288 |
| E-CTX-CIP-CS-3-12 | 94 | 375823 | 5122522 | 51.87 | 137875 | 11 | 0.00 | 264 |
| E-No drug-1-2 | 89 | 326339 | 5094581 | 51.88 | 139247 | 12 | 1.96 | 269 |
| E-No drug-1-19 | 77 | 536598 | 5090399 | 51.89 | 149900 | 9 | 0.00 | 254 |
| E-No drug-2-7 | 86 | 622466 | 5128228 | 51.88 | 149900 | 8 | 1.95 | 252 |
| E-No drug-2-12 | 89 | 375823 | 5101093 | 51.87 | 149900 | 11 | 0.00 | 276 |
| E-No drug-3-1 | 83 | 622466 | 5064213 | 51.88 | 170947 | 8 | 0.00 | 314 |
| E-No drug-3-5 | 82 | 386598 | 5035139 | 51.95 | 156005 | 9 | 0.00 | 360 |

**Table S2.** Sequence of sgRNA and primes about the selected target gene *arcA*

| Name | Sequence |
| --- | --- |
| sgRNA | GCTGACGGTACCCAGGTTCA |
| *arcA*-F | CTGGACGATCAGGCTTTG |
| *arcA*-R | GTAAGCATATTACGCTTCGC |
| *arcA*-HF | GCTGACGGTACCCAGGTTCAgttttagagctagaaatagcaag |
| *arcA*-HR | TGAACCTGGGTACCGTCAGCactagtattatacctaggactgag |
| *arcA*-UF | tgatatcgaattcctgcagcccgggCAACGAAATAGCGTTTCCAG |
| *arcA*-UR | TTAGTTGGCAATTTAGGTAGCAAACTTCGGCCCAGGCGCCAAACT |
| *arcA*-DF | GTTTGCTACCTAAATTGCCAACTAA |
| *arcA*-DR | tagaaGtagtggatcccccgggCGTATCTGTACTATTGCTGTTTATG |
| pSGKP-F1 | TCTCGTTTGGATTGCAACTG |
| xba-gRNA-R | GCCGCTCTAGAAGTAGTGGA |

**Table S3.** Characteristics of the deletions of 5 pJXP9-encoded ARG and the corresponding MICs among 6 transconjugants

| Plasmid type | Donors, Recipient, and Transconjugants | CTX | CS | FFC | FOS | CIP | NAL | Deletion gene |
| --- | --- | --- | --- | --- | --- | --- | --- | --- |
| - | 14028-rif | 0.5 | 1 | 2 | 4 | 0.015 | 4 | - |
| Ancestral plasmid | 14028-rif-pJXP9 | 128 | 16 | 256 | 512 | 0.125 | 32 | - |
| MDR ARGs loss | JE-CTX-CIP-1-6 | 256 | 8 | 8 | 512 | 0.125 | 32 | *floR* |
|  | JE-CTX-CS-2-2 | 128 | 16 | 256 | 512 | 0.06 | 4 | *oqxA*, *oqxB* |
| D1+D2 | JE-CIP-CS-2-6 | 128 | 16 | 1 | 256 | 0.015 | 1 | *floR*, *oqxA*, *oqxB* |
|  | JE-No drug-1-2 | 2 | 8 | 2 | 32 | 0.03 | 4 | *bla*_CTX-M-14_, *floR、fosA3*, *oqxA*, *oqxB* |
| D1 | JE-CTX-CIP-CS-2-5 | 128 | 16 | 128 | 256 | 0.03 | 2 | *oqxA*, *oqxB* |
|  | JE-No drug-2-7 | 256 | 8 | 256 | 256 | 0.03 | 4 | *oqxA*, *oqxB* |

**Table S4.** Summary of all amino acid substitutions in *arcA* from 36 endpoint-evolved 14028-pJXP9 clones from both mono-drug and combination-drug, as well as No-drug groups

| Mutation site | Number | Drug treatment |
| --- | --- | --- |
| L50Q | 1 | CTX-CIP (1) |
| M53I | 5 | CIP-CS (2), No drug (1), CIP-low (2) |
| P58T | 1 | CIP-high (1) |
| A66V | 4 | No drug (1), CIP-low (1), CTX-high (2) |
| A66T | 1 | CIP-high (1) |
| Q72* | 1 | CS-high (1) |
| E86K | 2 | CIP-CS (1), No drug (1) |
| D88N | 1 | CTX-CIP-CS (1) |
| G92S | 4 | CTX-CIP (2), CIP-high (1), CTX-high (1) |
| D98N | 2 | CTX-CS (2) |
| D99E | 1 | CTX-CIP (1) |
| D99N | 1 | CTX-CIP-CS (1) |
| P104L | 2 | CTX-CIP (2) |
| P104S | 2 | CIP-low (1), CS-low (1) |
| P104Q | 2 | CS-low (1), CS-high (1) |
| R108C | 1 | CIP-high (1) |
| P154L | 2 | CIP-low (2) |
| H171D | 1 | CTX-CIP-CS (1) |
| H171R | 1 | No drug (1) |
| T224I | 1 | CS-high (1) |
